# Linc00467 promotion of gastric cancer development by directly regulating miR-7-5p expression and downstream epidermal growth factor receptor

**DOI:** 10.1080/21655979.2021.1996014

**Published:** 2021-12-07

**Authors:** Li-Hao Deng, Hui Zhao, Li-Ping Bai, Jun Xie, Kai Liu, Feng Yan

**Affiliations:** a Department of Gastrointestinal Surgery, The Affiliated Zhongshan Hospital of Xiamen University; Institute of Gastrointestinal Oncology, Medical College of Xiamen University, Xiamen, China; b Medical Cosmetology, Xiamen Hospital of Traditional Chinese Medicine, Xiamen, China

**Keywords:** Gastric cancer, linc00467, miR-7-5p, epidermal growth factor receptor, proliferation, migration, invasion

## Abstract

Linc00467 is a vital regulator in tumor progression. This study explores the molecular mechanisms of linc00467 in gastric cancer (GC). Linc00467 expression was obtained and analyzed in GC tissue through exploration in the cancer genome atlas database. Then, real-time quantitative polymerase chain reaction was used to detect linc000467 expression in GC cells. Cell functions were observed using cell counting Kit-8, Transwell assay, Western blotting to testify the proliferation, migration, invasion, and the relative expression of epidermal growth factor receptor (EGFR) in GC cells. Moreover, a dual-luciferase reporter gene assay was used to verify the relationship between linc00467 and miR-7-5p. Results showed that the expression of linc00467 was overexpressed in GC. Linc00467 silencing decreased the GC cell proliferation, migration, and invasion. With mRNA verification and combined previous research, linc00467 directly regulated the miR-7-5p expression and downstream EGFR expression. Inhibited miR-7-5p could restore cell function, EGFR expression of GC cells when linc00467 knockdown occurs. Altogether, linc00467 directly regulates the miR-7-5p and EGFR signaling pathway to promote GC proliferation, migration, and invasion.

## 1. Introduction

Gastric cancer (GC) is the third most common cause of cancer death globally [1]. With the anticancer treatment, patients with early GC have been improved to 95% in the aspects of 5-year overall survival [2]. But GC is seldom diagnosed early for the lack of clinical manifestations and screening biomarkers. Effectiveness and strategy treatments were changed along with the tumor’s change, and most GC patients (>70%) developed to advanced-stage while care was been provided, where some patients were diagnosed with metastasis, which causes a poor overall prognosis [3]. Thus, diagnosis and targeted therapy of GC in time has become the crucial approach in influencing the prognosis of GC patients.

Molecular mechanisms driving GC tumorigenesis, progression, and metastasis need to be elucidated in order to improve the methods of the early diagnosis and find new therapeutic targets of GC. Several long noncoding RNAs (IncRNAs) were identified due to the development of molecular biology technology. LncRNAs are transcripts longer than 200 nucleotides, transcribed by RNA polymerase II. Accumulated evidence showed that lncRNAs are novel regulators of gene expression at different levels, including chromatin modification, transcription, and post-transcription [4,5]. LncRNAs share a common function of shape gene expression by titrating microRNAs (miRNAs), and this phenomenon was named competing endogenous RNA (ceRNA) [6,7]. For example, lncRNA H19, a 2.7-kb lncRNA, its ceRNA activities that modulate several miRNAs/genes aixes, including miR-20a-5p/transforming growth factor beta receptor 2, miR-216a−3p/actin alpha 2, miR-21/programmed cell death 4, and miR-19a/inhibitor of DNA binding 2, etc [8–11]. Simultaneously, elevated H19 expression is found in many cancer types, and therefore can be a potential therapeutic target in cancer [12]. Besides, several lncRNAs, such as nuclear enriched abundant transcript 1, growth arrest-specific 5, and metastasis-associated lung adenocarcinoma transcript 1, are well studied and known to play critical regulatory roles in diverse processes in cancer [6]. Moreover, lncRNAs are also biomarkers for screening, prediction of response to treatment, prognosis, and monitoring of cancer progression [13]. Previous studies have shown that in GC, lncRNA Hox transcript antisense intergenic RNA is implicated in tumorigenesis and progression, and lncRNA gastric cancer-associated transcript 2 is a diagnostic marker [14]. However, these are one part of lncRNAs. And more studies are needed to investigate other lncRNAs roles in GC.

Linc00467 is a novel lncRNA with a function that is still not fully understood. Previous neurological research has shown that N-Myc plays a suppressive role in linc00467 gene transcription, and decreased linc00467 downstream protein-coding gene RD3 mRNA expression while promoting dickkopf WNT signaling pathway inhibitor 1 expression, all these changes reduced neuroblastoma cell survival [15]. Linc00467 was highly expressed in lung adenocarcinoma and plays a promotional role in tumorigenesis through dickkopf WNT signaling pathway inhibitor 1 knockdown to activate Wnt/β-catenin axis [16]. Another research has shown that linc00467 served as a ceRNA of miR-107 to promote cervical cancer progression and maybe a novel potential therapeutic target [17]. Linc00467 was significantly overexpressed in several cancers. However, individual studies have found that there are differences. Contrary to other studies, digestive system cancer research showed that linc00467 expression was decreased in hepatocellular carcinoma (HCC) and contributed to the inhibition role in cell viability, proliferation, migration, and invasion through the miR-9-5p/peroxisome proliferator-activated receptor alpha signaling pathway [18].

As cell signaling pathways are involved in different stages of cancer, including initiation, progression, and metastasis, lncRNAs participates in all aspects of tumorigenesis via these pathways. Thus, it is necessary to conduct research in this area. According to The Cancer Genome Atlas (TCGA) data, linc00467 expression in GC tissue is significantly higher than that in the adjacent tissues. Therefore, we speculated that linc00467 also plays an important role in GC development. However, previous studies regarding digestive system tumors and linc00467 have inconsistent results, and little is known about the role of linc00467 in GC. This study focused on the function and potential mechanism of linc00467 in GC. We detected the expression of linc00467 in GC cell lines, revealed its abnormal expression in GC cells. Further, we knockdown linc00467 by small interfering RNA (siRNA), and explored the potential targeting molecules as well as the pathways mediated by linc00467 to reveal the function of linc00467 in GC cells. The results of this study provided new clues for further study.

## 2. Materials and methods

### 2.1 Cell culture

Five GC cell lines, including MKN45, HGC-27, NCI-N87, AGS, and MKN28, together with normal human gastric epithelial cells (GES-1), were purchased from American Type Culture Collection (Manassas, VA, USA). GC cell lines (MKN28, MKN45, and NCI-N87) were cultured in Roswell Park Memorial Institute (RPMI) 1640 Medium (Guangzhou Cellcook Cell Biotechnology, LTD, Guangdong, China) supplemented with 10% fetal bovine serum (FBS) (Thermofisher Scientific, Waltham, MA, USA). HGC-27 was cultured in minimum Eagle’s medium (Gibco, Grand island, LY, USA) containing 10% FBS. AGS and GES-1 were cultured in F12K (CellCook, Guangzhou, China) containing 10% FBS. All the above cells were kept in a humidity incubator with 5% CO_2_ at 37°C.

### 2.2 Cell transfection

When cell densities reached 90%, 30 pmol siRNA was transfected into cells using 2-μl Lipofectamine 2000 (Invitrogen, Carlsbad, CA, USA) following the manufacturer’s instructions. In our study, siRNA, including si-linc00467, siRNA-negative control (si-NC), miR-7-5p inhibitor, and inhibitor NC, were designed and synthesized by GenePharma (Shanghai, China). Sequences are as follows: si-linc00467 sense (5ʹ-3ʹ): GCUGGCAAAUAUGAAGGUATT; antisense (5ʹ-3ʹ): UACCUUCAUAUUUGCCAGCTT. si-NC sense (5ʹ-3ʹ): UUCUCCGAACGUGUCACGUTT; antisense (5ʹ-3ʹ):ACGUGACACGUUCGGAGAATT. miR-7-5p inhibitor (5ʹ-3ʹ): AACAACAAAAUCACUAGUCUUCCA; inhibitor NC (5ʹ-3ʹ): CAGUACUUUUGUGUAGUACAA.

### 2.3 Cell proliferation assay

Cell proliferation assay was conducted in line with previous studies with few amendments [19] . In brief, after transfection for 48 h, cells were digested and replaced into a 96-well plate at a density of 5 × 10^3^ per well in 100-μl medium. After 24 h, 48 h, and 72 h, 10-μl cell counting Kit-8 (CCK-8, Beyotime Biotechnology, Shanghai, China) per well was added and incubation for another three hours, then 450-nm spectrophotometric value was detected using Elx800 (BioTek, Winooski, Vermont, USA).

### 2.4 Transwell assay

In our study, a 24-well Transwell chamber was used to detect migration and invasion. In brief, cells (1 × 10^4^) in serum-free medium were plated in the upper chamber, and 10% FBS medium was plated in the lower chamber. Furthermore, cells were kept in an incubator (37°C, 5% CO_2_) for 48 h. The migrating cells were fixed with 4% paraformaldehyde (Solarbio, Beijing, China), after which 0.1% crystal violet was used for dyeing. Notably, before conducting the invasion assay, 10-μl Matrigel (diluttion: 1:3; BD Biosciences, Shanghai, China) should be precoated in the upper chamber. Finally, the stained migration and invasion cells were counted under an optical microscope (Olympus, Tokyo, Japan). The invaded/migrated cells were counted in five random fields for each well, and the relative invaded/migrated rate was calculated relative to the si-NC group.

### 2.5 Dual-luciferase reporter gene assay

Firstly, miRanda was used to find the binding site between linc000467 and miR-7-5p. Secondly, the sequences of linc000467 wild type (linc000467-WT) and linc000467 mutant type (linc000467-MUT) were synthesized and cloned into pmirGLO plasmid (Promega, Madison, WI, USA) in GenePharma. Negative control of miR-7-5p mimics (NC mimics) and miR-7-5p mimics was also provided by GenePharma. Thirdly, linc000467-WT (or linc000467-MUT) and NC mimics (or miR-7-5p mimics) were co-transfected into cells for 48 h using Lipofectamine 2000. Finally, cells were collected to conduct luciferase assay following the instruction of dual-luciferase reporter assay system (Promega). And the relative luciferase activity was calculated following previous studies [19].

### 2.6 Real-time quantitative polymerase chain reaction (RT-qPCR)

RT-qPCR was used in this study to evaluate the relative expression of linc00467, EGFR, miR-7-5p, miR-26a-5p, miR-494-3p, miR-200b-3p, and miR-29 c-3p. In brief, total RNA was extracted using TRIzol reagent (Invitrogen, Carlsbad, CA, USA) following provider’s guide. Reverse transcription (2-μg total RNA) was conducted using RevertAid RT Reverse Transcription Kit (Takara, Tokyo, Japan). SYBR Green Premix kit (Takara,) was used for PCR detection. The reaction conditions were 95°C for 5 min, followed by 40 cycles of 95°C for 30 s and 68°C for 1 min, 72°C for 2 min. Sequences of primers used in RT-qPCR are listed in Table 1.

**Table 1.** Sequences of primers used in this study

Primer Name	Sequences (5ʹ-3ʹ)
LINC00467-F	CAGCACCGATCCCGACATAG
LINC00467-R	CTGGCTTCCTGAAGACGATGA
EGFR-F	AGGCACGAGTAACAAGCTCAC
EGFR-R	ATGAGGACATAACCAGCCACC
GAPDH-F	GAGTCAACGGATTTGGTCGT
GAPDH-R	GACAAGCTTCCCGTTCTCAG
miR-7-5p-F	GCGTGGAAGACTAGTGATTT
miR-7-5p-RT	GTCGTATCCAGTGCAGGGTCCGAGGTATTCGCACTGGATACGACACAACA
miR-26a-5p-F	AAGCGACCTTCAAGTAATCCAG
miR-26a-5p-RT	GTCGTATCCAGTGCAGGGTCCGAGGTATTCGCACTGGATACGACAGCCTA
miR-494-3p-F	CGTTGAAACATACACGGGA
miR-494-3p-RT	GTCGTATCCAGTGCAGGGTCCGAGGTATTCGCACTGGATACGACGAGGTT
miR-200b-3p-F	AACACGCTAATACTGCCTGGT
miR-200b-3p-RT	GTCGTATCCAGTGCAGGGTCCGAGGTATTCGCACTGGATACGACTCATCA
miR-29 c-3p-F	TAGCACCATTTGAAATCG
miR-29 c-3p-RT	GTCGTATCCAGTGCAGGGTCCGAGGTATTCGCACTGGATACGACTAACCG
Universe-R	GTGCAGGGTCCGAGGT
U6-F	CTCGCTTCGGCAGCACA
U6-R	AACGCTTCACGAATTTGCGT

### 2.7 Western blot assay

In our study, we used a Western blot assay to assess the relative EGFR protein expression. Total protein was isolated using RIPA Lysis Buffer (Thermofisher Scientific). Protein was separated using 10% sodium dodecyl sulfate-polyacrylamide gel electrophoresis, and then was transferred onto a polyvinylidene fluoride membrane. After that, the membrane was blocked using 5% fat-free milk for one hour and then incubated with primary antibodies anti-EGFR (dilution: 1:5000; boster, #PB0039) and anti-glyceraldehyde-3-phosphate dehydrogenase (dilution: 1:8000; Proteintech, #60,004-1-lg) overnight at 4°C. After that, the membrane was incubated into the horseradish peroxidase-conjugated secondary antibody (dilution: 1:5000; boster, #BA1056) for two hours at room temperature. Electro-Chemi-Luminescence method was used to detect the bands. The signal was visualized via a gel imaging (Odyssey, LI‐COR Biosciences) detection system. Finally, the ImageJ software (National Institutes of Health, Bethesda, MD, USA) was used to calculate the densitometric of the bands.

### 2.8 Statistical analysis

Data were exhibited as mean ± standard deviation, and GraphPad Prism software v7.0 was used for statistical testing (GraphPad Software, San Diego, CA, USA). Differences between groups were analyzed using nonparametric Student’s t-test, p-values were calculated, and p < 0.05 was considered statistically significant. All experiments were repeated thrice. Moreover, We obtained the data related to gene expression and prognosis of linc00467 from the Gene Expression Profiling Interactive Analysis (GEPIA website: http://gepia.cancer-pku.cn/detail.php?gene=linc00467). We utilized miRanda to predict target miRNA. The network regulation diagram was drawn using Cytoscape.

## 3. Results

Linc00467 has been reported play an important role in lung adenocarcinomal, cervical cancer, hepatocellular carcinoma. However, it’s role in the GC is unclear now. According to TCGA data, linc00467 expression in GC tissues is significantly higher than that in the adjacent tissues. We then detected the expression of linc00467 in GC cell lines, revealed its abnormal expression in GC cells. Further, we knockdown linc00467 by siRNA, and found the ability of proliferation, migration, and invasion have been inhibited. Combined with previous research and further verification, we found linc00467 directly regulated the miR-7-5p and EGFR expression. Inhibited miR-7-5p could restore cell function and EGFR expression of GC cells when linc00467 knockdown occurs. Altogether, linc00467 directly regulates the miR-7-5p and EGFR signaling pathway to promote GC proliferation, migration, and invasion.

### 3.1 Overexpression of linc00467 in gastric cancer

**Figure 1.**
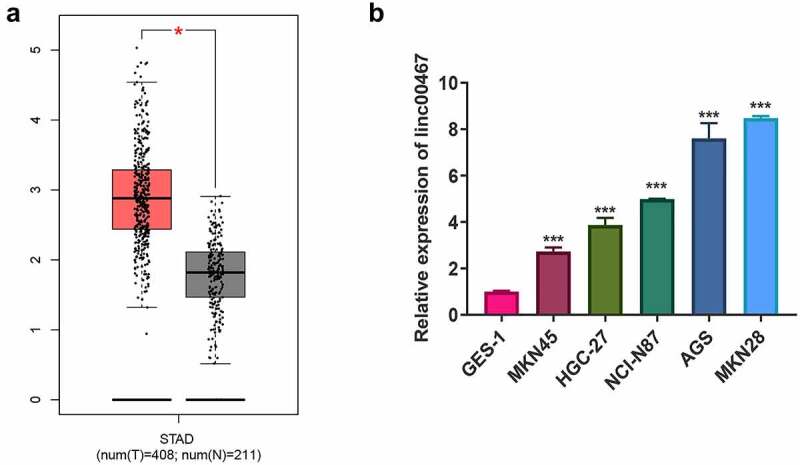
Linc00467 is overexpressed in gastric cancer tissues and cells. (a) the expression of linc00467 in GC tissues from the the cancer genome atlas database. N = 211. (b) the relative expression of linc00467 in MKN45, HGC-27, NCI-N87, AGS, MKN28, and GES-1 was determined by real-time quantitative polymerase chain reaction. *, indicates p < 0.05; ***, indicates p < 0.001

According to TCGA data, the expression of linc00467 in GC tissue is significantly higher than that in adjacent tissues (Figure 1(a)). In further exploration, we detected the expression of linc00467 in GC cells by RT-qPCR. The result showed that the relative expression of linc00467 in GC cells (including MKN45, HGC-27, NCI-N87, AGS, and MKN28) was upregulated about 2–8 fold compared with control GES-1 (Figure 1(b)). In which, compared to GES-1, the expression of linc00467 was upregulated about 7–8 fold, so we selected them for further study.

### 3.2 Silence of linc00467 inhibits cell function of proliferation, migration, and invasion

**Figure 2.**
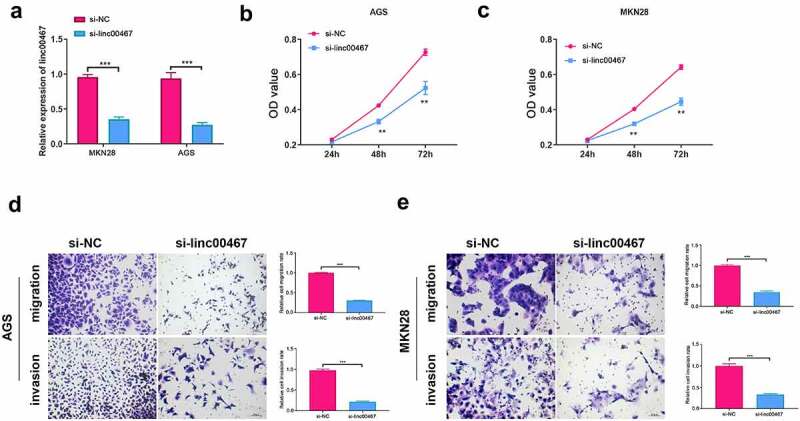
Knockdown of linc00467 can inhibit cell proliferation, migration, and invasion. (a) the relative expression of linc00467 in MKN28 and AGS cell lines after si-linc00467 was transfected for 48 h was determined using real-time quantitative polymerase chain reaction. (b) and (c): After linc00467 was knocked down, cell proliferation was examined in AGS or MKN28 cell lines at 24 h, 48 h, and 72 h by cell counting kit-8. (d) and (e): Migration and invasion of AGS or MKN28 cells were determined by transwell assay after si-linc00467 was transfected for 48 h. ***, indicates p < 0.001, scale bar = 25 μm, magnification: 200 ×

To further study the function of linc00467, we silence the expression of linc00467 to observe changes in cell proliferation, migration, and invasion. We first verified the interference effect of siRNA. According to the Figure 2(a) results, the relative expression of linc00467 was significantly down-regulated (about 2-fold) after si-linc00467 was transfected into cells for 48 h compared with the si-NC group. This verified the effectiveness of si-linc00467 in this study. As shown in Figure 2(b,c), AGS or MKN28 cell proliferation capacity was significantly down-regulated at 48 h and 72 h in si-linc00467 group compared to the si-NC group, respectively. Additionally, silencing linc00467 could suppress migration and invasion of AGS and MKN28 cells above 2-fold compared to the si-NC group (Figure 2(d,e)). These results have demonstrated that silencing linc00467 could inhibit the proliferation, migration, and invasion of GC cells.

### 3.3 Bioinformatics analyzed of linc00467 and verification

**Figure 3.**
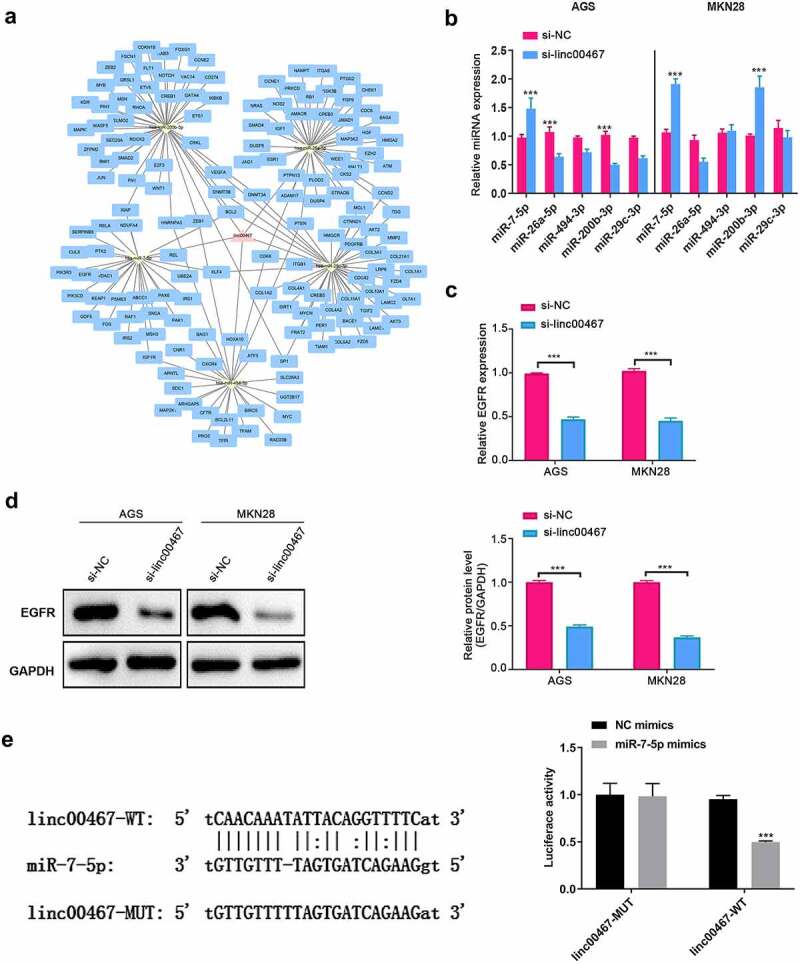
Bioinformatics analyzed linc00467 targeted miRNAs and mRNAs, and preliminary screening. (a) bioinformatics analysis and prediction of target miRNAs that linc00467 may adsorb via miRanda. (b) the relative expressions of miR-7-5p, miR-26a-5p, miR-494-3p, miR-200b-3p, and miR-29 c-3p were tested using real-time quantitative polymerase chain reaction after si-linc00467 was transfected for 48 h in AGS or MKN28 cell lines. (c) The relative expression of epidermal growth factor receptor (EGFR) was detected by real-time quantitative polymerase chain reaction after si-linc00467 was transfected for 48 h in AGS or MKN28 cell lines. (d) EGFR protein expression was examined using western blot after si-linc00467 was transfected for 48 h. (e) dual-luciferase reporter gene assay was used to detect the binding site between linc000467 and miR-7-5p. ***, indicates p < 0.001

Because lncRNA may play a role through the ceRNA mechanism, we have conducted bioinformatics analysis to explore the miRNA and mRNA that linc00467 may regulate and made a network regulation diagram (Figure 3(a)). We selected and verified 5 miRNAs and their target mRNA among them. As shown in Figure 3(b), in AGS cells, miR-7-5p was significantly upregulated (about 1.5 fold) in the si-linc00467 group compared to the si-NC group; while in MKN28 cells, both miR-7-5p and miR-200b-3p were significantly upregulated in the si-linc00467 group compared to the si-NC group (about 2-folds), which probably indicated that miR-7-5p is one of the target miRNAs of linc00467. A previous study reported that EGFR, as a target gene of miR-7-5p, was significantly suppressed at mRNA and protein levels by miR-7-5p, inhibiting GC development and progression [19]. Therefore, we further detected the expression of EGFR. Results demonstrated that the gene and protein expressions of EGFR were all significantly down-regulated (about 2-fold) in si-linc00467 groups compared to the si-NC group, as shown in Figure 3(c,d). Additionally, we confirmed that linc000467 can directly bind with miR-7-5p using dual-luciferase reporter gene assay, as the luciferase activity was significantly down-regulated in the linc00467-WT + miR-7-5p mimics group relative to the linc00467-WT + NC mimics about 2-fold (Figure 3(e)). Moreover, a previous study confirmed that EGFR also can directly bind with miR-7-5p [19]. Therefore, these results indicated that linc00467 probably affected GC progression via directly regulating miR-7-5p and EGFR expression.

### 3.4 Linc00467 promoted gastric cancer cell proliferation, migration, and invasion by regulating the miR-7-5p and EGFR signaling pathway

**Figure 4.**
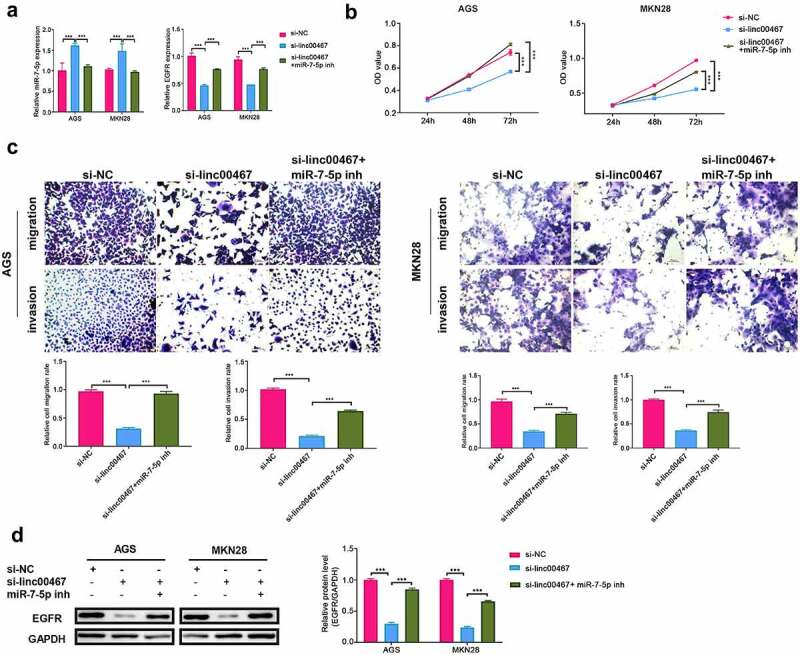
Linc00467 promoted miR-7-5pproliferation, migration, and invasion of gastric cancer cells by regulating miR-7-5p and EGFR signaling pathways. (a) The relative expression of miR-7-5p and EGFR was determined by real-time quantitative polymerase chain reaction in si-NC, si-linc00467, si-linc00467 + miR-7-5p inhibitor groups. (b) The proliferation of AGS and MKN28 cells was assayed using cell counting kit-8 in si-NC, si-linc00467, si-linc00467 + miR-7-5p inhibitor groups, respectively. (c) migration and invasion of AGS and MKN28 cells were determined by transwell assay in si-NC, si-linc00467, si-linc00467 + miR-7-5p inhibitor groups, respectively. Scale bar = 25 μm, magnification: 200 × . (d) The protein expression of EGFR was measured by western blotting in si-NC, si-linc00467, silinc00467 + miR-7-5p inhibitor groups. *** p < 0.001

To verify whether linc00467 contributes to GC progression by regulating the miR-7-5p and EGFR signaling pathway, we knockdown linc00467 and miR-7-5p simultaneously in AGS and MKN28 cells. As shown in Figure 4(a), miR-7-5p was significantly upregulated (about 1.5-fold), and EGFR was down-regulated (about 2-fold) in the si-linc00467 group compared to those in the si-NC group. However, transfection with si-linc00467 and miR-7-5p inhibitor simultaneously, the EGFR expression was significantly reversed compared with the si-linc00467 group (Figure 4(a)). These indicated that miR-7-5p inhibitors could promote the expression of EGFR. Moreover, results also showed that cell proliferation, migration, and invasion of AGS and MKN28 cells were inhibited in si-linc00467 group compared to those in si-NC group (Figure 4(b,c)). While knockdown linc00467 and miR-7-5p simultaneously reduced inhibition effect on cell proliferation, migration, and invasion caused by interference with linc00467 (Figure 4(b,c)). Western blotting results showed that the expression of EGFR was down-regulated (above 2-fold) after knockdown linc00467 compared to those in the si-NC group (Figure 4(d)). Furthermore, EGFR expression is upregulated relative to the expression of the interfering linc00467 group after treatment with both miR-7-5p inhibitor and si-linc00467 (Figure 4(d)). These results indicated linc00467 could promote proliferation, migration, and invasion by directly regulating miR-7-5p and EGFR signaling pathway in GC cells.

## 4. Discussion

Among the previous studies, little was known about linc00467 in GC cells. In this study, we found that linc00467 was overexpressed in GC cells and promoted the cell function of GC cells, including proliferation, migration, and invasion, via miR-7-5p and EGFR signaling pathway.

Previous studies have demonstrated that linc00467 was abnormally expressed (most were upregulated, individual decreased) in various types of malignant tumors, which contribute to tumorigenesis through different kinds of signaling pathways, such as miR-9-5p/peroxisome proliferator activated receptor alpha, miR-20b-5p/cyclin D1, and DNA methyltransferase 1/p53 [18,20,21]. In the studies of gastrointestinal tumors, Zengyao Li et al. reported that the level of linc00467 was overexpressed in colorectal cancer (CRC) tissues and cell lines, and the expression of ferritin light chain was positive-relevant to the activation of linc00467/miR-133b axis in CRC, which further leads to CRC cell chemoresistance and promoted CRC metastasis [22]. Presently, we have not consulted the study on linc00467 in GC. Regarding research of linc00467 in digestive system tumors, besides CRC, most of them focused on HCC. These studies also demonstrated that linc00467 upregulated in HCC tissues and cells, performing diverse functions in tumorigenesis, metastasis, and cancer progression [23–26]. Previous studies have reported that linc00467 promotes cell proliferation and metastasis through insulin-like growth factor-2 messenger RNA-binding protein 3/tumor necrosis factor receptor-associated factor 5, miR-18a-5p/neural precursor cell expressed developmentally down-regulated 9, miR-509-3p/platelet-derived growth factor receptor alpha pathways, indicating linc00467 signaling may impede apoptosis, and contribution to axitinib resistance of HCC, these results indicated that linc00467 may serve as a promising biomarker and target for HCC treatment [23,25,26]. However, different from linc00467 upregulation in cancer cells, a study by Kerui Cai et al. reported that linc00467 was aberrantly decreased in HCC, especially in metastases [18]. Further analysis of the author indicated that linc00467 can directly regulate the miR-9-5a expression and downstream target peroxisome proliferator activated receptor alpha signaling [18]. Additionally, a study regarding esophageal carcinoma also found that upregulated linc00467 improved cell proliferation while inhibiting cell apoptosis by regulating the axis of miR-485-5p/dolichyl-phosphate N-acetylglucosaminephosphotransferase 1 [27]. Research regarding linc00467 outside the digestive system in malignant tumors mainly focused on lung cancer and glioma. It was also demonstrated that the expression of linc00467 was elevated in lung adenocarcinoma tissues and cell lines, involved in tumorigenesis [20,28,29]. Several initiated signaling pathways, including linc00467/miR-20b-5p/cyclin D1 pathway [20] and STAT1-induced upregulation of linc00467 promoted lung adenocarcinoma progression through epigenetically suppressing dickkopf WNT signaling pathway inhibitor 1 to activate Wnt/β-catenin signaling [16]. Regarding glioma, studies have demonstrated that linc00467/DNA methyltransferase 1/p53 pathway, and linc00467/miR-200a/E2F transcription factor 3 pathway might be involved in the occurrence of glioma [21,30]. In this study, we have further discovered that linc00467 was significantly upregulated in GC cell lines compared with control GES-1. The GC cell lines tested includes MKN45, HGC-27, NCI-N87, AGS, and MKN28. Especially in AGS and MKN28 cell lines, the overexpression was more significantly higher. These results were consistent with most of the previous studies of cancer. Different from the above verified signaling pathways, in this study, we reported a new-found signaling of linc00467 in GC that linc00467 regulate the proliferation, migration, and invasion of GC cells via miR-7-5p and EGFR signaling pathway.

Previous studies have verified that miR-7-5p is not only a tumor suppressor but also reverses drug resistance in certain cancers [31,32]. To explore the potential signaling pathway and molecular function of linc00467, we predicted the target miRNA of linc00467 uaing miRanda database. We selected miRNAs according to the number of target binding sites between linc00467 and miRNA together with the reports of miRNA functions in GC. Among these miRNAs, the miR-7-5p, miR-26a-5p, miR-494-3p, miR-200b-3p, and miR29c-3p were further verified. Results showed that only miR-7-5p correlated with the expression of linc00467 in AGS and MKN28 cell lines. Moreover, dual-luciferase reporter gene assay confirmed that linc00467 could directly regulate miR-7-5p. Additionally, miR-7-5p significantly decreased tumor cell viability, invasion ability, and migration ability in GC [19]. These results indicate that linc00467 directly regulates miR-7-5p expression, which contributes to GC proliferation, migration, and invasion.

Some target genes of miR-7-5p have been identified in human diseases. A study by Haiping Xiao reported that miR-7-5p regulates target neuro-oncological ventral antigen 2 (NOVA2) expression to suppress tumor metastasis of non-small cell lung cancer (NSCLC), and when NOVA2 was overexpressed, the miR-7-5p-mediated inhibitory effect on lung cancer cells was reversed [33]. And Qin Li et al. reported that P21-activated kinase 2 was also a direct target gene for miR-7-5p in NSCLC [34]. The Chang-You Yin et al. reported that overexpression of special AT-rich sequence-binding protein 1, a direct target gene of miR-7-5p, attenuates the miR-7-5p-mediated inhibitory effect on cell migration and invasion in glioblastoma [35]. Additionally, miR-7-5p has been reported to directly bind with EGFR to inhibit the migration and invasion of GC [19]. Therefore, we selected this gene as the downstream gene of miR-7-5p for further study.

EGFR is frequently mutated and/or overexpressed in cancers, and is considered the target of cancer therapies in clinical practice [36]. Registry databases of miRNA, including miRDB v21, TargetScan 7.2, miRWalk 3, and miRTarBase 7.0, found 134 miRNAs were predicted to be potential regulators of EGFR. In this study, we found EGFR was significantly down-regulated when linc00467 was knockdown in GC cells, while the miR-7-5p inhibitor could reverse the situation. The ability of proliferation, migration, and invasion had the same changes as EGFR expression. These results indicate that linc00467 promotes the proliferation, migration, and invasion of GC cells by regulating the expression of miR-7-5p and EGFR signaling pathway. This was consistent with another previous study on GC by Zichang Yang et al., which demonstrated miR-7-5p could bind to specific sites of urothelial cancer associated 1 to regulate the target EGFR through ceRNA function [37]. Combing these results, we thought linc00467 could directly regulate miR-7-5p and EGFR signaling pathways to participate in GC proliferation, migration, and invasion, which may provide new clues for further GC experiments.

However, more studies are needed. For example, whether miR-7-5p regulates other target gene in GC progress; in vivo studies are needed to confirm the conclusion of this research as evidence that in vivo animals would strengthen the conclusions. Additionally, more GC tissue samples are still needed to further verify the clinical significance of linc00467 and the conclusions.

## 5. Conclusion

In conclusion, this study showed that linc00467 was upregulated in GC, and promoted GC cell proliferation, migration, and invasion by directly regulating the expression of miR-7-5p and its downstream target EGFR. These results also provided new insight for further study, as linc00467 may be a potential biomarker of GC patients and a potential molecular target for GC cancer therapy.

## Data Availability

The data that support the findings of this study are available from the corresponding author by reasonable request.
